# Health risks of Rohingya refugee population in Bangladesh: a call for global attention

**DOI:** 10.7189/jogh.08.020309

**Published:** 2018-12

**Authors:** Mohammad Mainul Islam, Tasmiah Nuzhath

**Affiliations:** Department of Population Sciences, University of Dhaka, Dhaka, Bangladesh

In Bangladesh, more than 836 000 Rohingya refugee population is in need of humanitarian assistance [1]. These refugees faced discrimination in their native land in terms of various restrictions imposed on them due to the effective denial of their citizenship. This led to several human rights violations including limited access to health care services [2]. Currently they are under significant health risks and it has become a challenge to address their health needs. Due to the increasing number of Rohingya refugees and their congested living conditions in camps, there has been an overwhelming increase in their health risks [3]. Refugees and affected community require 9 million liters of safe water daily, and water, sanitation and hygiene (WASH) services are reaching only 30% of the Rohingya people in need. Thus leaving them with no other option than to fetch dirty water from muddy streams [4]. 85% of the refugees still have no access to latrines [5]. All of which in turn increases the risk of communicable disease outbreak [4]. There has been reports of measles outbreak amongst new arrivals, the number of cases reported is 419 [6]. The largest oral cholera vaccination was held in the refugee camps and even though it was able to reach 100% of the targeted population, the risks of waterborne and other infectious diseases are still exceptionally high due to their unhygienic living conditions [7]. Diphtheria outbreak has resulted in 38 deaths and more than 5800 suspected cases of diphtheria have been reported as of February 2018 [8]. There have also been reports on respiratory problems and skin diseases among the refugees who have arrived since 25^th^ August-with 10 846 and 3422 cases respectively [9].

Among the refugees, 720 000 are children [4]. 14 740 orphan Rohingya children have been identified since September 20, 2017 in the settlements in Ukhia and Teknaf [10]. An estimated 250 000 children under the age of 8 require life-saving interventions through community-based activities such as vaccination campaigns whereas 240 000 children under-five years need malnutrition prevention and treatment support through nutritious supplementary food.16 965 children with severe acute malnutrition (SAM) require inpatient and outpatient treatment. 204 000 adolescent girls need nutritional support and 237 500 children from 6 months to 15 years need to receive measles-rubella (MR) vaccine [11].

**Figure:**
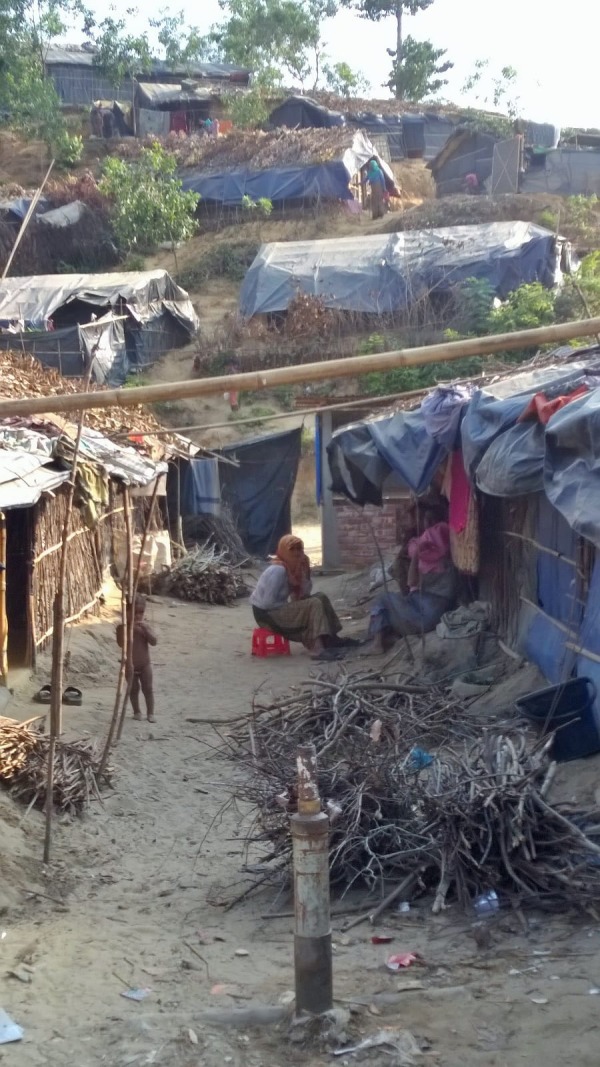
Photo: Some of the shelters at Kutupalong camp site for the Rohingyas in Cox's Bazar (from the collection of Helena Derwash, used with permission)

In the refugee camps, 54% of the Rohingya are below the age of 18; 52% are women with 23% of them between the ages of 18 and 59 years [12]. Among 91 556 adolescent girls and women, 54 633 are pregnant or lactating mothers [4]. Lactating mothers (9.2% of total refugees) and pregnant women (4.9% of the total population) have been identified as the two highest numbers of vulnerable group within the Rohingya Refugees [13]. As of 22^nd^ October, an estimated 42 000 pregnant women, 72 000 lactating mothers and 240 000 under-five children need health assistance [11]. Majority of women are giving births at home, and only 22% of births occur in health facilities [14]. 2592 lactating women and 1145 pregnant women have been admitted for malnutrition treatment [5]. They are also among the first to experience additional barriers in accessing the scarce and overstretched humanitarian relief services. Furthermore, not only are they among the most affected groups but are also usually the last to be consulted (if at all) about their needs and provided with the least information about where and how to claim relief services [10]. 120 000 pregnant and lactating mothers require prevention and treatment from malnutrition through nutritious supplementary food [11]. Even though both Myanmar and Bangladesh have low prevalence of HIV among the South Asian countries, however the Rakhine state had the highest prevalence of HIV in 2015. In addition to this, the current predicament makes the victims of sexual violence more predisposed to the risks and transmission of HIV [15]. There have also been 21 cases of HIV patients reported among the refugees until Oct 8, 2017 [16].

There is an inadequate supply of essential reproductive along with maternal, child and new-born health services. Furthermore, there is insufficient clinical management of rape survivors, family planning as well as adolescent friendly health services, especially in the provision of these services in hard-to-reach areas. Moreover, there are no extensive HIV and TB services, although there have been cases of HIV reported among the refugees [17]. There is limited accessibility to inpatient as well as secondary health services which also includes referral system and quality of care and health care services implemented at the settlement lack standardization [8]. Overcrowded settlements and the rapid influx of refugees challenge the ability of service providers to identify private and safe services for women. There is incessant new influx of refugees which leads to overburdening of the existing facilities like WASH or health facilities and thus services are still not available and accessible to many of the refugees. The sheer size, density and unplanned nature of the make-shift settlements hosting refugees remain a major obstacle to setting up the communal infrastructures necessary to coordinate services at site level [17].

Mental health impact on the forcibly displaced refuges are significant. Refugees are reported to suffer from the flashback of the massacre, anxiety, acute stress, recurring nightmares, sleep deprivation, eating or even speaking disorder [18]. Methodical rape on women and girls and violent deaths of family members have compounded the mental health situation of the survivors of this physical violence. Women and children reported facing sexual violence including gang rapes which resulted in vaginal tears, infections and posttraumatic disorders [19]. There has been increase in the incidence of sexual violence among the refugees in Bangladesh which was exacerbated by the unavailability and low quality of post-rape care services [20]. From the end of August 2017 to the end of February 2018, MSF has treated 226 survivors of sexual violence at MSF’s Sexual and Reproductive Health Units, out of which 162 of them were rape survivors. Majority of the survivors were below 18 years [21]. Children face the danger of long-term psychological and social distress [22]. Since refugees are dependent on the humanitarian assistance for their survival and struggle daily for food assistance, this acts as a stressor for majority of them as well [23].

In addition, the overall situation and health risks will be exacerbated when the monsoon season arrives as flooding will adversely affect the latrines, tube wells and health facilities built in the camps [24]. The international community and Bangladesh government need to address the vulnerability of these refugees by giving humanitarian and financial assistance to them. There is need to scale up health services and increase access to essential reproductive health and child newborn care, especially for Rohingyas living in hard-to-reach areas. Community health workers need to be effectively trained to ensure adequate health promotion, promotion of hygiene and home visits to pregnant women. Scaling up of mental health service provision in primary health care settings is needed. Information needs to be adequately provided to the refugees. Furthermore, in the case of epidemics, rapid response is necessary and to ensure that reliable health statistics remain paramount. Thus, organizations need to give more attention to the collection and dissemination of data. As refugees, their condition has aggravated because of limited financial aids and overcrowded unhealthy living conditions in settlements and camps. All of which will exacerbate their access to health care services, predisposing them to numerous health risks and increase the chance of disease outbreak. Thus along with the government, private sectors and international communities must collaborate to assist the refugees in their dire condition for the improvement of their health status.

## References

[1] Intersector Coordination Group. Situation Update: Rohingya Refugee Crisis Cox’s Bazar. Intersector Coordination Group; 2018.

[2] Watch HR. World Report 2017. Available: http://www.hrw.org/world-report/2017. Accessed: 30 October 2017.

[3] Intersector Coordination Group. WASH Sector Cox’s Bazar-Situation Report. Intersector Coordination Group; 2017.

[4] United Nations Children’s Fund. Outcast and Desperate: Rohingya refugee children face a perilous Future. New York: UNICEF; 2017.

[5] United Nations High Commissioner for Refugees. Operational Update-Bangladesh. Geneva: UNHCR; 2017.

[6] United Nations Children’s Fund. Bangladesh Humanitarian Situation report-10 (Rohingya Influx). New York: UNICEF; 2017.

[7] International Organization for Migration. Bangladesh (Rohingya Influx) Health Situation Report. Geneva: IOM; 2017.

[8] World Health Organization. Weekly Situation Report. Bangladesh: WHO; 2018.

[9] United Nations High Commissioner for Refugee. Disease threatens refugees in Bangladesh in unplanned sites. Geneva: UNHCR; 2017.

[10] Women UN. Gender Brief on Rohingya Refugee Crisis Response in Bangladesh. New York: UN Women; 2017.

[11] United Nations Children’s Fund. Bangladesh Humanitarian Situation report-8 (Rohingya Influx). New York: UNICEF; 2017.

[12] Refugee Relief and Repatriation Commissioner. RRRC Fact Sheet-Family Counting. Bangladesh: Refugee Relief and Repatriation Commissioner; 2017.

[13] International Organization for Migration. Needs and Population Monitoring Round 6 Assessment Report-Cox’s Bazar, Bangladesh. Geneva: International Organization for Migration; 2017.

[14] United Nations Population Fund. UNFPA Rohingya Humanitarian Response. New York: UNFPA; 2018.

[15] World Health Organization. Bangladesh/Myanmar: Rakhine Conflict 2017. Public Health Situation Analysis and Interventions. Geneva: WHO; 2017.

[16] DBC News. 21 Rohingas are HIV affected. *2017**. **Available**:* https://www.dbcnews.tv/paper/910. Accessed: 30 October 2017.

[17] Intersector Coordination Group. Situation Report: Rohingya Refugee Crisis Cox’s Bazar. Intersector Coordination Group; 2017.

[18] Jazeera A. The mental health toll of the Rohingya crisis. 2017. Available:https://www.aljazeera.com/indepth/features/2017/10/mental-health-toll-rohingya-crisis-171010111603004.html. Accessed: 20 March 2018.

[19] Watch HR. Failing Rohingya Rape Victims in Bangladesh: Refugees from Burma Lack Access to Crucial Services. 2018. Available:https://www.hrw.org/news/2018/02/23/failing-rohingya-rape-victims-bangladesh. Accessed: 20 March 2018.

[20] Inter-Agency Working group on Reproductive Health Crises. Women and girls critically underserved in the Rohingya humanitarian response. Inter-Agency Working group on Reproductive Health Crises; 2018. 26203479

[21] Médecins Sans Frontières. Bangladesh: Activities update on Cox's Bazar. Geneva: MSF; 2018.

[22] Bangladesh WV. Psychological support for refugee children of Myanmar in Bangladesh. Bangladesh: World Vision Bangladesh; 2018.

[23] World Health Organization. Health Sector Bulletin: Rohingya Refugee Crisis in Cox’s Bazar. Geneva: WHO; 2018.

[24] United Nations High Commissioner for Refugee. UNHCR warns monsoons in Bangladesh could put protection of Rohingya refugees at serious risk. Geneva: UNHCR; 2018.

